# The Truman Show for protozoan parasites: A review of in vitro cultivation platforms

**DOI:** 10.1371/journal.pntd.0009668

**Published:** 2021-08-26

**Authors:** Smita Sutrave, Martin Heinrich Richter

**Affiliations:** German Federal Institute for Risk Assessment (BfR), Department of Biological Safety, Berlin, Germany; Centro de Pesquisa Gonçalo Moniz-FIOCRUZ/BA, BRAZIL

## Abstract

Protozoan parasites are responsible for severe disease and suffering in humans worldwide. Apart from disease transmission via insect vectors and contaminated soil, food, or water, transmission may occur congenitally or by way of blood transfusion and organ transplantation. Several recent outbreaks associated with fresh produce and potable water emphasize the need for vigilance and monitoring of protozoan parasites that cause severe disease in humans globally. Apart from the tropical parasite *Plasmodium* spp., other protozoa causing debilitating and fatal diseases such as *Trypanosoma* spp. and *Naegleria fowleri* need to be studied in more detail. Climate change and socioeconomic issues such as migration continue to be major drivers for the spread of these neglected tropical diseases beyond endemic zones. Due to the complex life cycles of protozoa involving multiple hosts, vectors, and stringent growth conditions, studying these parasites has been challenging. While in vivo models may provide insights into host–parasite interaction, the ethical aspects of laboratory animal use and the challenge of ready availability of parasite life stages underline the need for in vitro models as valid alternatives for culturing and maintaining protozoan parasites. To our knowledge, this review is the first of its kind to highlight available in vitro models for protozoa causing highly infectious diseases. In recent years, several research efforts using new technologies such as 3D organoid and spheroid systems for protozoan parasites have been introduced that provide valuable tools to advance complex culturing models and offer new opportunities toward the advancement of parasite in vitro studies. In vitro models aid scientists and healthcare providers in gaining insights into parasite infection biology, ultimately enabling the use of novel strategies for preventing and treating these diseases.

## Author summary

In light of the far-reaching social and economic repercussions of communicable, zoonotic parasitic diseases on human health, it is imperative to continue to strive toward developing in vitro models for in-depth scrutiny and understanding of pathogenicity, as well as for innovations toward combating these infections. This review, to our knowledge, is the first to offer a qualitative summary of the existing models for culturing protozoan parasites with major relevance to human health in vitro. The present work aims to provide a reference guide on the current state of in vitro culture of these protozoan parasites and offers a foundation to facilitate exchange of expertise among researchers, clinicians, and healthcare workers. This comprehensive review would aid in enabling discussions on new intervention approaches to fill in the knowledge gaps in the field of parasitic diseases affecting the global population.

## Introduction

The availability and proper functioning of sanitation services are intricately linked to maintaining public health standards; however, this still remains to be achieved in all parts of the world [1–3]. Despite major strides made in improving early childhood health in recent years, the mortality rate for the age group under 5 years in part due to parasite-related diseases remains highly correlated with geographical location, i.e., low- and middle-income countries [4,5]. Tropical and subtropical climate zones with endemic insect vectors, lower standards of living, lack of access to basic amenities such as clean drinking water, and public sanitation facilities as well as areas with political instability show a higher burden of disease (BoD) associated with parasitic diseases [6]. However, parasitic disease outbreaks have also been reported in well-developed regions of the world, as shown by the outbreak of *Cryptosporidium hominis* after heavy flooding in Germany [7], a foodborne outbreak of *Cryptosporidium parvum* in Norway [8], and several outbreaks involving both species in the United Kingdom in recent years [9].

In the past decades, several governmental and nongovernmental organizations have been involved in implementing programs aimed toward prevention and treatment of life-threatening protozoan diseases [10–12]. Treatment options have been developed for some of these parasitic diseases such as malaria, the leading cause of parasitic disease-related deaths worldwide, but are not always readily available. The malarial parasite, *Plasmodium* spp., is a prime example of a protozoan receiving continued funding and scientific interest. However, other protozoan diseases such as Chagas disease and African trypanosomiasis have largely been neglected despite their severe disease profiles [13].

Additional protozoan parasites add to the range of severe and debilitating diseases. Young children and immunocompromised individuals are at risk for *C*. *parvum* infections, while *Toxoplasma gondii* infections during pregnancy pose serious risks to the unborn child [14,15]. *Naegleria fowleri*, an emerging acarpomyxean parasite known to thrive in warm water bodies, causes primary amebic encephalitis associated with rapid deterioration of patient health and a high fatality rate [16]. This disease is now being reported in regions with no prior cases, implicating climate change as an important driver for spread of the pathogen.

Well-established in vitro models provide a valuable alternative to in vivo models by giving in-depth information on life cycle stages as well as circumventing the ethical issues of laboratory animal use. Another major advantage of in vitro models is the ready availability of parasites for further experiments without having to be passaged through an animal host. Factors challenging the culture of parasites in the laboratory include complexity of parasitic life cycles including several life cycle stages, asexual and sexual cycles needing specific, and varied hosts depending on the parasite in question. As with all in vitro models, one of the main concerns is in vitro–in vivo correlation. The more complex a parasitic life cycle, the more challenging this correlation becomes, proving to be one of the main hindrances in the progression of parasite in vitro culture. New technologies such as 3D tissue and organoid models, 3D tissue printing, hollow fiber technologies, and other novel technology approaches have provided well-founded hope to develop successful and correlating in vitro systems that are of particular interest for parasitic disease research.

The current review is not a systematic listing of all publications on the vast topic of in vitro models for the maintenance of protozoan parasites relevant to human health. However, this review aims to provide a comprehensive overview of available in vitro models for protozoa listed as significant agents of morbidity and mortality in humans by WHO and CDC. To our knowledge, this is the first review summarizing in vitro models of protozoan parasites with a significant BoD. The protozoan parasites have been classified into 3 categories, members belonging to the Apicomplexan group, the trypanosomes, and other protozoans, respectively.

Table 1 outlines the disease burden of protozoan parasites worldwide including number of cases, fatality rate, and treatments available for each protozoan parasite included in this review. Only parasitic diseases for which an in vitro system has been described in literature have been outlined in the table. Table 1 includes the disability-adjusted life year (DALY) metric that has been used by WHO Global Health Estimates (GHE) to measure the global burden of disease (GBD) on society due to various ailments starting in 2000 with the latest data published in 2016 [17]. For any given disease, one DALY is equivalent to one lost year of “healthy” life and is calculated by summing up the years of life lost (YLL) due to premature death and the years lived with disability (YLD) due to suboptimal health caused by that specific disease [18].

**Table 1:** List of protozoans and the associated diseases for which in vitro models are published.

	Endoparasite	Disease caused	Transmission stage	Primary mode of transmission	Treatment	Cases worldwide	Disease related deaths	DALY Score
**Apicomplexa**	*Cryptosporidium* spp.	Cryptosporidiosis	Thick-walled oocysts	Fecal–oral, contaminated soil and food and water	Nitazoxanide	64,003,709 [19]	27,553 [19]	2,159,331 [19]
	*Toxoplasma gondii*	Toxoplasmosis	Oocysts in cat feces, tissue cysts in meat	Cat feces, contaminated soil, undercooked meat containing tissue cysts, congenital transmission, blood transfusion or organ transplantation	Pyrimethamine in combination with sulfadiazine and folinic acid	20,710,906 acquired [19]	0 acquired [19]	1,153,779 acquired [19]
						98,900 congenital [19]	1,409 congenital [19]	526,515 congenital [19]
	*Plasmodium* spp.	Malaria	Sporozoites	Bite of infective female *Anopheles* mosquitoes	Hydroxychloroquine, Artemisinin-based combination therapies	228,000,000 [20]	446,446 [21]	37,368,766 [21]
**Flagellates**	*Trypanosoma cruzi*	Chagas disease (American trypanosomiasis)	Trypomastigotes	Feces of triatomine insects, contaminated food, congenital transmission, blood transfusion or organ transplantation	Abenznidazole, Nifurtimox	8,000,000 [22]	7,728 [21]	252,204 [21]
	*Trypanosoma brucei*	African sleeping sickness	Metacyclic trypomastigotes	Bite of infective tsetse flies, congenital transmission or blood transfusion	Fexinidazole, Nifurtimox in combination with eflornithine	977 [23]	3,077 [21]	203,497 [21]
	*Leishmania* spp.	Visceral, cutaneous and mucocutaneous leishmaniasis	Promastigotes	Bite of infective female phlebotomine sandflies	Liposomal amphotericin B, meglumine antimoniate	n.a.	14,454 [21]	1,068,537 [21]
	*Giardia duodenalis*	Giardiasis	Cysts	Contaminated food and water, person-to-person contact	Metronidazole, Tinidazole, Fexinidazole	183,842,615 [19]	0 [19]	171,100 [19]
**Amoebas**	*Entamoeba histolytica*	Amebiasis	Mature cysts	Contaminated food and water, person-to-person contact	Metronidazole, Tinidazole	103,943,952 [19]	5,450 [19]	515,904 [19]
	*Naegleria fowleri*	PAM	Trophozoites	Nasally from contaminated fresh water bodies and recreational facilities	Amphotericin B, Miltefosine, medically induced hypothermia	310 [24]	Over 97% fatality rate [25]	n.a.

DALY, disability-adjusted life year; n.a., data not available; PAM, primary amebic meningoencephalitis.

## Literature search criteria

Searches of PubMed using the search terms “in vitro model,” “in vitro culture,” and “in vitro cultivation” along with the scientific name for each parasite were used to identify the references cited in this review. Titles and abstracts were screened for recent advances and innovations in the field of in vitro culture for the 9 protozoan parasites included in this review.

## Review of existing in vitro models for protozoan parasites

### Apicomplexa

Most apicomplexan parasites are characterized by the presence of a plastid-like organelle known as the apicoplast [26]. The apicoplast plays a role in various metabolic pathways including fatty acid synthesis that are vital for the members of Apicomplexa [27]. This group encompasses a diverse range of organisms displaying complex life cycles including both asexual and sexual reproductive phases [28]. In recent years, numerous publications have focused on in vitro models for apicomplexan parasites especially *Cryptosporidium*, *Toxoplasma*, and *Plasmodium*.

#### *Cryptosporidium* spp.

Unlike other members of the Apicomplexan group of parasites, organisms of the genus *Cryptosporidium* spp. lack the apicoplast [29]. *C*. *parvum* and *C*. *hominis* are capable of causing severe diarrhea and gastrointestinal discomfort in humans, which can be life threatening in young children, the elderly, and immunocompromised individuals [30]. The disease has also major implications for life stock as it frequently causes fatal diarrhea in calves [31]. The infective oocysts when ingested establish a disease cycle within the host, which propagates itself into the surrounding environment through the shedding of oocysts in stool [32]. *C*. *parvum* is a monoxenous parasite, completing its life cycle in a single host. The asexual cycle includes multiplication of the parasite by schizogony, followed by the sexual cycle involving the production of environmentally stable thick-walled oocysts shed in feces and thin-walled oocysts causing autoinfection of host intestinal epithelium [28].

*Cryptosporidium* is by far the most well-researched parasite in terms of the abundance and complexity of culture models described. Previously, various human and animal cell lines have been the basis for monolayer cultures of *C*. *parvum*. However, complete development including production of sexual stages has been challenging in monolayers [32]. In recent years, various research groups have attempted to establish advanced in vitro culture models especially for *C*. *parvum*, summarized for better comparison in Table 2. The table provides at a glance the methods used as well as various advantages and disadvantages of the in vitro systems. As evident from the approaches described in the table, in spite of the large variety of in vitro models for long-term cultivation of *C*. *parvum* established so far, there is a lack of consensus among researchers regarding culture methods, as well as choice of media, and host cell lines used.

**Table 2:** Recent advances in the field of in vitro culture systems for *Cryptosporidium parvum*.

	Nikolaev and colleagues (2020) [35]	Wilke and colleagues (2019) [36]	Josse and colleagues (2019) [39]	Heo and colleagues (2018) [38]	Miller and colleagues (2018) [40]	Baydoun and colleagues (2017) [37]	DeCicco RePass and colleagues (2017) [34]	Morada and colleagues (2016) [41]
**System**	Mini-gut tubes derived from mouse intestinal organoids	Stem-cell derived ALI monolayer with intestinal epithelial spheroids at 37°C with 5% CO2 in incubator	Bioreactor using COLO-680N cell lines	3D organoid culture system with duodenal biopsies; microinjection of parasite suspension into organoids	2D COLO-680N cell culture platform	Colonic explant as 3D culture model, humidified incubator at 37°C with 5% CO2	3D silk scaffolds model 37°C with 5% CO2	3D culture using 200-μm polysulphone hollow fibers with MEMSS medium (MEM plus supplemensts and serum) pumped through the hollow fibers
**Host cell lines**	Mouse intestinal crypts	Ileal IECs from mice expanded and maintained as 3D spheroid cultures in Matrigel	COLO-680N esophageal cancer cell line	Small intestinal organoids established using duodenal biopsies from healthy human donors	COLO-680N esophageal cancer cell line	Mouse colonic explants	Colon cancer cell lines Caco-2 and HT29-MTX	Colon cancer cell line HCT-8
***C*. *parvum* strains**	Iowa	AUCP-1 isolate from calves	Moredun and Iowa	Iowa	Moredun and Iowa	Iowa	Iowa	Iowa
**Media**	Advanced DMEM/F12 medium supplemented with 1× Glutamax, 10 mM HEPES and 100 μg ml−1 penicillin–streptomycin	50% L-WRN CM with 10 um Y-27632 ROCK inhibitor, medium changed every 2 days, cells passaged every 3 days	RPMI-1640 with L-glutamate, FBS, penicillin-streptomycin, medium exchange weekly	Wnt-rich culture medium Advanced DMEM/F12	RPMI-1640 medium supplemented with 10% FBS, 100 U/mL of penicillin, 100 μg/mL of streptomycin and 250 ng/mL of amphotericin B	HEPES buffered DMEM/F12, 10% FBS, penicillin, streptomycin, L-glutamine, insulin/transferrin/selenite mix, keratinocyte growth factor (KGF) etc. medium exchange every 48 hours	modified DMEM with 10% fetal bovine serum, transferrin, penicillin, streptomycin, amphotericin	MEM plus supplements and serum
**Infectivity assay**	None	Oocysts infective to mice	None	SPF mice infected with broken organoids	None	None	None	Immunosuppressed and immunocompromised mouse models
**Duration of maintenance of culture**	4 weeks	20 days postinfection	Two weeks (standard culture flasks) and up to 4 months (2-week break recommended between harvests)	Continuous culture maintained for 28 days	Cultures remained viable and produced oocysts for 8 weeks without sub-culturing	Up to 35 days	Continuous culture for 15 to 17 days	Continuous culture for >6 months
**Life cycle stages**	Complete life cycle including all sexual and asexual stages	Sexual and asexual stages documented	Sexual and asexual stages documented	Parasite propagates and completes its life cycle in organoids. Oocysts generated are infectious to mice	Oocysts and other non-extra-cellular stages produced	Parasite completed life cycle and produced oocysts	Asexual, sexual stages, formation of oocysts, capable of supporting complete life cycle	In vitro production of oocysts which are infectious to mice
**Techniques for detection and quantification of parasite**	Immunofluorescence assay and TEM imaging	Immunofluorescence microscopy, electron microscopy, and qRT-PCR	Western blot with L23-A antibodies and immunofluorescence using L23-A and PCR	qRT-PCR, electron microscopy, and immunofluorescence staining (Sporo-Glo)	qPCR of 18S rRNA Crypt-a-Glo, Sporo-Glo staining, SEM, and AFM for oocysts, lipidomics using MALDI-TOF	Real-time qRT-PCR of 18S rDNA and fluorescence immunoassay	Immunofluorescence assay, confocal microscopy, SEM and qRT-PCR of 18S rRNA	Real-time qRT-PCR of 18S rRNA, fluorescently labeled antibodies, polyamine analysis, enzyme assays and SEM
**Advantages**	Luminal accessibility and ability of the system to support long-term host–microorganism cocultures; the scaffold is permeable to gases, nutrients, and macromolecules facilitating adhesion, proliferation, and differentiation of intestinal stem cells	ALI supports robust long-term growth of parasite (100-fold), complete life cycle development, production of infective oocysts	COLO-680N provides a good culture platform: readily infected, cells remain fit, supports full cycle, long-term cell survival	Versatility of use for multiple purposes including parasite development, host–parasite interaction, drug screening	Sustainable, continuous propagation of infective oocysts on a small-scale in standard tissue culture with readily available equipment, easy to implement, drug discovery, parasite biology	In vivo -like microenvironment, parasite-induced neoplasia, host–pathogen interactions, carcinogenesis, easy monitoring of experiments, standardization, and reproducibility	Complete life cycle development, host–parasite interaction, drug targets, genetically modified parasites	Complete life cycle, large surface area for metabolite/gas exchange, biphasic medium mimicking the gut with redox and nutrient control, high output of oocysts, long-term host–parasite interaction, pharmacokinetic data, study of growth factors
**Disadvantages**	Lack of host immune components	Lack of host immune response	Intracellular *C*. *parvum* count in COLO-680N is lower than in HCT-8 cells, membrane disruption issues in the mini-PERM bioreactor exist	The number of parasites decreased over time, number of oocysts formed within organoids smaller than in host animals, requires microinjection of organoids	Missing 3D culture aspect	Animal experiments required for harvesting colonic explant tissue	Specialized equipment needed to pump nutrients and remove waste, model does not show that new oocysts are functionally comparable to normal oocysts, low number of oocysts produced, not conducive for host–parasite analysis or rapid drug screening	Specialized equipment and knowledge needed; expensive medium supplements, not scalable and not amenable for investigation of individual stages and assessing host–parasite interaction
**Novelty**	Homeostatic mini-intestines through scaffold-guided organoid morphogenesis	ALI platform, identification of a new stage called “late stage microgamont,” in vitro genetic crosses	Easy to handle, low-cost bioreactor system using COLO-680N lines; western blot targeting 60S ribosomal subunit L23-A for semi-quantitative analysis (17 kDa)	Organoids derived from healthy human tissue mimic 3D tissue structure and function; contain epithelial cells which enables study of host epithelium–parasite interaction; a compromise between single cell culture and in vivo models	Novel use of COLO-680N cells to produce more oocysts for subsequent infection than HCT-8 models, 50-fold increase at 10 days, cryopreservation system for long-term storage and successful resuscitation of parasites	3D culture system from SCID mice instead of immunocompetent mice	3D human intestinal model, *C*. *parvum* passaged from infected to fresh scaffold for 3 cycles, depth-graded oxygen profiles favorable for *C*. *parvum*	Hollow fiber technology, biphasic model simulating gut microenvironment

ALI, air–liquid interface; CM, conditioned medium; IEC, intestinal epithelial cell; qRT-PCR, quantitative reverse transcription polymerase chain reaction; SPF, specific-pathogen-free; TEM, transmission electron microscopy.

Prior attempts at in vitro cultivation of *C*. *parvum* utilizing monolayers and axenic cultures have been marked by low counts of infective oocysts and incomplete life cycle with only asexual development reported [32]. The in vitro models highlighted in Table 2 offer high oocysts yields, long-term maintenance of culture and production of all parasitic stages. However, some of these models have innate disadvantages, such as the need for specialized equipment and media components as well as extensive knowhow and expertise required in implementation [33,34]. In addition, some of the in vitro models require mouse intestinal tissues [35–37] or donor biopsies [38] as basis for the model. Apart from ensuring viability of parasites for infection studies, robustness, and scalability of cultures, these advanced models are stepping-stones between cell monolayers and in vivo studies. However, there is ongoing contention in the scientific community as to whether these in vitro systems truly support uninterrupted maintenance of the life cycle due to the difficulty of direct microscopic visualization of the parasites within the various models and the fact that these results are yet to be corroborated by other research laboratories [29]. New combinations of existing technologies employing select features of current models such as simplified protocols, lower maintenance costs in terms of equipment and media, and inclusion of host immunological aspects are imperative for mass production of parasitic stages and continued infection of subsequent cultures, thereby decreasing the use of animal models in the future.

#### Toxoplasma gondii

*T*. *gondii*, the causative agent of toxoplasmosis, has a worldwide distribution with most infections being mild or asymptomatic [28]. However, exposure to the parasite via consumption of raw meat and contact with contaminated soil or cat feces during pregnancy leads to a range of severe complications for the unborn child [30]. Felids are the definitive hosts for *T*. *gondii*; however, the parasite has the ability to infect all warm-blooded animals including humans [31]. The sexual cycle of *T*. *gondii* involving the production of micro- and macrogametocytes occurs exclusively in the intestinal epithelial cells of felids, while the asexual cycle involving repeated cycles of endodyogeny can take place in all mammalian cells of the indefinitive hosts such as humans [28].

In vitro cell culture methods are well established for *T*. *gondii*, with various studies published using several distinct cell lines most commonly using tachyzoite stages for infection [42–44]. Alternatively, other *T*. *gondii* stages such as oocyst-derived sporozoites or tissue cyst–derived bradyzoites may be used in experimental infection models [45]. The following features make *T*. *gondii* an ideal protozoan for infection studies: existence of several transmission stages, ease of cultivation in most vertebrate cells, availability of established animal models, and feasibility of genetic crosses. However, further insights into pathogenesis have been hindered by the absence of models simulating the architectural complexity of the host intestinal epithelium [46].

A recent study revolutionized the field of *T*. *gondii* research by successfully carrying out the sexual cycle in vitro using mouse intestinal organoids via bradyzoite infection [47]. This was achieved by making use of the fact that members of the feline family lack the enzyme delta-6-desaturase responsible for breaking down linoleic acid. Inhibiting the activity of this enzyme along with dietary supplementation of linoleic acid was conducive to the completion of the sexual cycle of *T*. *gondii* parasites in mice.

A recent study reported for the first time coinfection with *T*. *gondii* type 1 RH strain tachyzoites and *Giardia duodenalis* WB6 (ATCC 50803) trophozoites in murine organoid-derived monolayers [46]. By simplifying culture conditions, this study outlined protocols for the establishment and maintenance of 3D intestinal organoids and organoid-derived monolayers for 4 animal hosts with immediate relevance to the study of host–parasite interactions for gastrointestinal parasites.

**Fig 1:**
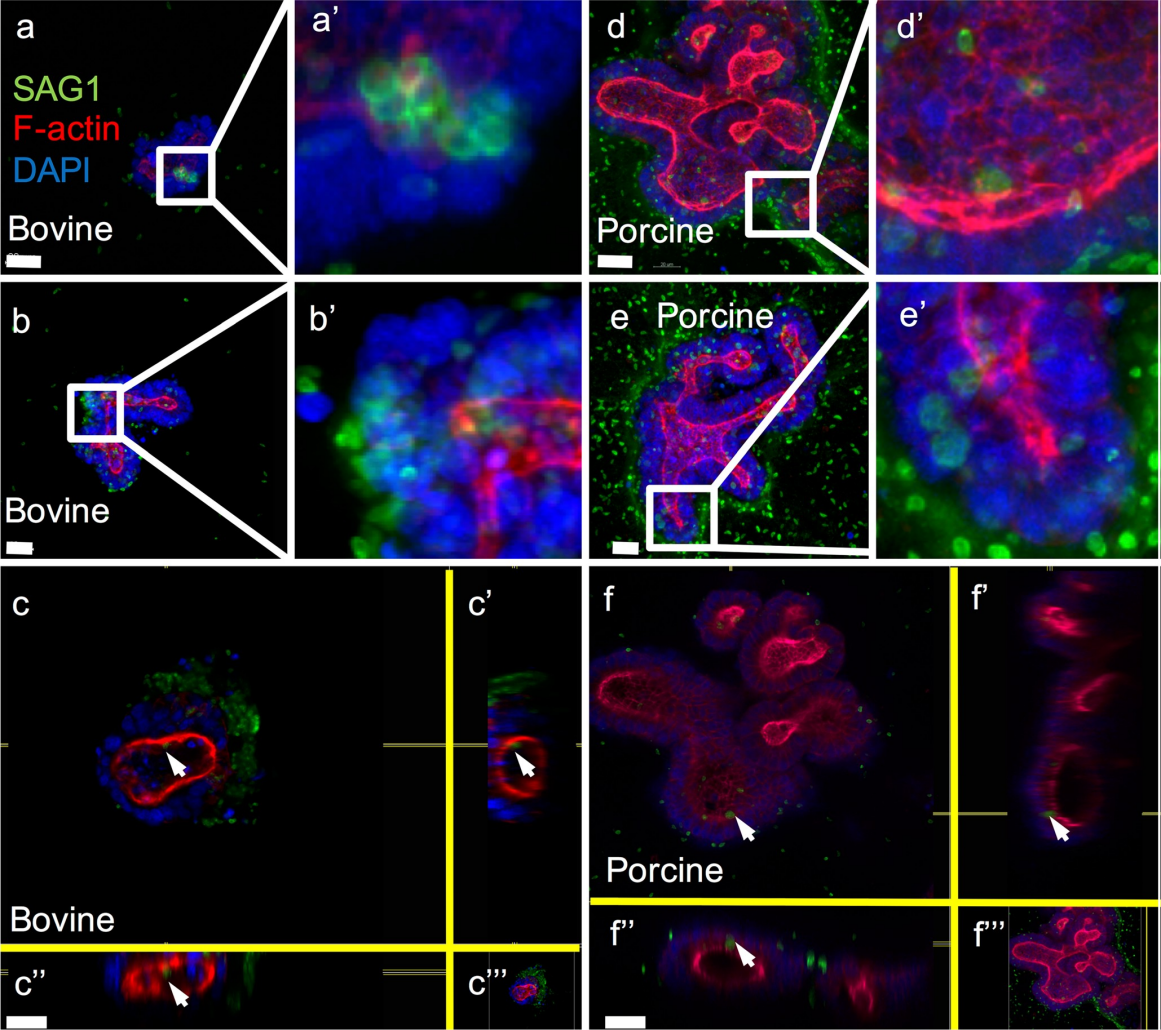
Schematic illustration of organoids using parasite surface antigen SAG1. Schematic illustration showing immunofluorescent staining of bovine **(a–c)** and porcine **(d–f)** intestinal organoids using parasite surface antigen SAG1 (Alexa Fluor 488—green), F-actin (rhodamine—red), and nuclei (DAPI—blue). Organoids were incubated with 10^6^ *T*. *gondii* RH strain tachyzoites for 1 hour at 37°C, embedded in Matrigel and incubated for a further 24 hours at 37°C. SAG1 in green shows intraluminal parasites (a, d, a’, and d’), parasite rosettes indicating replication (b, e, b’, and e’), and white arrows point to intracellular parasites (c, c’, c”, f, f’, and f”). Scale bars represent 20 μm in panels a, b, d, and e and 30 μm in panels c and f. 400x magnification. Open-access image reused from [48].

Previously, Derricott and colleagues [48] developed 3D intestinal organoids using porcine and bovine jejunum as seen in Fig 1. The porcine and bovine organoids incubated with 1 × 10^6^ *T*. *gondii* RH strain tachyzoites for 1 hour, then cultured in Matrigel for 24 hours showed distinct foci of infection, thus proving their susceptibility to *T*. *gondii* infection. The use of such intestinal samples offers a viable alternative and limits the need for animal experiments. The investigators showed that the intestinal crypts thus isolated and cultured into organoids in Matrigel, could be cryopreserved and subsequently resuscitated with 75% to 80% viability. This model offers a feasible long-term solution for obtaining renewable culture material independent of the use of animal models.

#### *Plasmodium* spp.

A considerable amount of interest and funding has been devoted to studying malaria and its causative agent *Plasmodium* spp., enabling new breakthroughs toward management of the leading cause of mortality due to a parasite worldwide [30]. Malaria, transmitted by species of *Anopheles* mosquitoes, is characterized by relapse of the disease where the dormant stages known as hypnozoites become reactivated in liver tissues [28]. Sporozoites introduced by the mosquito into the host bloodstream undergo exoerythrocytic schizogony in liver cells, giving rise to mature schizonts that in turn release merozoites. These merozoites then undergo erythrocytic schizogony producing schizonts, which, upon maturation, release merozoites and continue further asexual multiplication. After ingestion by the mosquito vector via a blood meal, merozoites develop into male and female gametocytes and continue the sexual cycle in the insect midgut [31]. Previously, large-scale production of erythrocytic stages was achieved by 2 methods both using RPMI 1640 medium with 25mM HEPES buffer: continuous flow and petri dish–candle jar systems. Early cultivation experiments of exoerythrocytic stages utilized either cultures of infected rat liver cells or infection of embryonic fibroblasts with sporozoites [49].

A recent groundbreaking study provided unequivocal proof of the reactivation of hypnozoites in vitro by utilizing a transgenic *Plasmodium cynomolgi* dual fluorescent reporter line; for this purpose, the construct pCyCEN_Lisp2mCherry_hsp70_GFP (Addgene, ID 137169) was created [50]. Dormant hypnozoites were green-fluorescent protein positive and mCherry negative, while the developing forms were positive for both fluorescent reporters. Recent studies have reported parasite enrichment and short-term culture of *Plasmodium vivax*; however, the lack of a robust long-term culture system has hindered progress in areas such as transcriptomics for these parasites [51].

**Fig 2:**
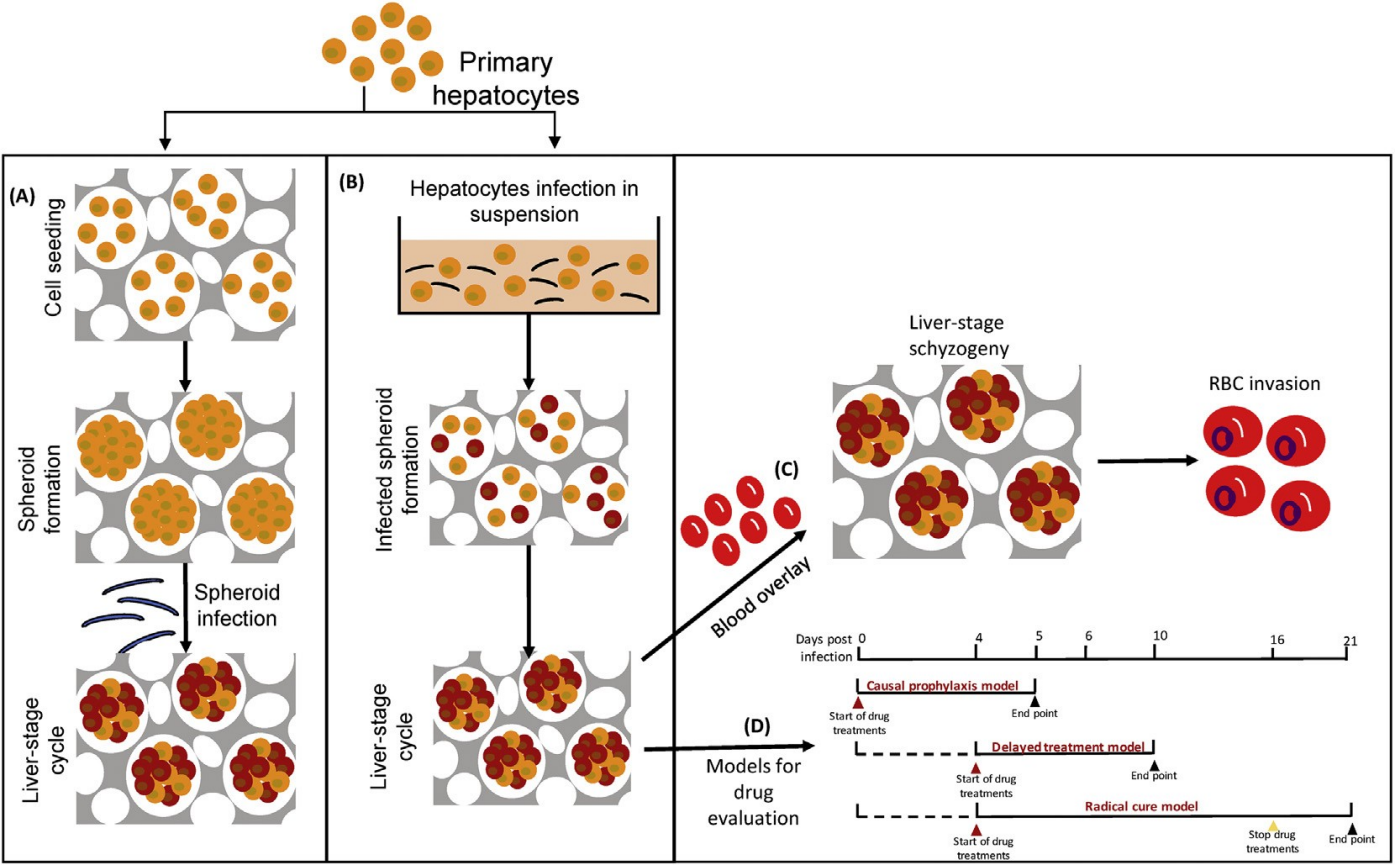
In vitro hepatic spheroid formation. Schematic diagram showing hepatic spheroid formation in the 3D Cellulosponge in vitro system for *P*. *cynomolgi* parasites. **(A)** Spheroids of uninfected hepatocytes. **(B)** Spheroids of hepatocytes pre-infected using sporozoites. **(C)** Successful reinfection and completion of life cycle in erythrocytes. **(D)** Potential models for drug evaluation in liver stages are shown. Open-access image reused from [52]. RBC, red blood cell.

Chua and colleagues [52] published a 3D hepatic spheroid model using Cellulosponge (Invitrocue, Singapore) to study *P*. *cynomolgi*. The 3D model was successful in producing liver stages within the hepatocytes embedded in the sponge (Fig 2). This model is the first successful platform closely resembling *P*. *cynomolgi* in in vivo studies. Using this hepatic spheroid system, the complete liver stage life cycle including sporozoite invasion and merozoite release leading to new infection was achieved. The current model using *P*. *cynomolgi* parasites could also be employed to study life cycle and mode of infection in other *Plasmodium* species.

### Flagellates

Members belonging to the family Trypanosomatidae are flagellated, parasitic protozoans causing severe diseases in humans and are transmitted by insect vectors. They cause 3 severe diseases: American trypanosomiasis also known as Chagas disease, African trypanosomiasis, and leishmaniasis. *G*. *duodenalis* is a flagellated intestinal parasite causing giardiasis in humans.

#### Trypanosoma cruzi

*Trypanosoma cruzi* is the causative organism of Chagas disease, also known as American trypanosomiasis. Originally confined to South America, this crippling disease is transmitted by direct contamination of a bite wound with feces or urine of the triatomine bug (*Triatoma* spp.) or via the alimentary tract by consumption of contaminated food [30]. In humans, *T*. *cruzi* exists as rounded amastigotes in muscles and nerve cells and as trypomastigotes in peripheral blood. Amastigotes undergo binary fission and transform into promastigotes, followed by epimastigote forms and are then released into the blood stream as trypomastigotes [28]. Increased international travel has led to the spread of the disease outside its endemic area [53]. Without proper treatment, the chronic phase of the disease can last a lifetime. According to WHO, it is not feasible to eradicate *T*. *cruzi* due to the extensive reservoir of the parasites in the wild animal population in endemic regions. In the future, climate change may play an increasing role in the spread of the vector to other parts of the globe.

There have been several publications on 2D in vitro cultures using monolayers for *T*. *cruzi* where both trypomastigote and amastigote stages were easily cultivated in Eagle’s Minimum Essential Medium (MEM) with 2% fetal bovine serum; however, separation of the stages to obtain pure cultures was challenging [49]. A 3D culture using cardiomyocyte spheroids was detailed by Garzoni and colleagues to study cardiomyopathy caused by *T*. *cruzi* [54]. Cardiac cells obtained from mouse embryos were plated in agarose coated 96-well plates, enabling the formation of 3D cardiac microtissues. Following infection by *T*. *cruzi*, an increase in both area and volume of the infected cardiac microtissues was observed in the spheroids.

Another recent publication sheds light on the transmigration of *T*. *cruzi* trypomastigotes by employing a 3D spheroid model mimicking infection conditions in situ [55]. Apart from the added aspects of cell-to-cell interactions and the presence of an extracellular matrix (ECM), which are both missing in monolayer cultures, a unique feature of this system is the direct visualization of host and parasite cells by differential labeling with 2 fluorescent dyes. The 3D HeLa cell spheroids were labeled with the red fluorescence protein (RFP) and the trypanomastigotes were labeled with carboxyfluorescein succinimidyl ester (CFSE) to visualize transmigration of trypomastigotes within the spheroids using confocal microscopy. A follow-up study by Rodriguez and colleagues [56] published in 2020 showed that *T*. *cruzi* trypomastigotes were less infective in HeLaR2 spheroids than in 2D monolayers and concluded that 3D spheroids provide an optimal physiological environment for studying host–parasite interactions.

#### Trypanosoma brucei

*Trypanosoma brucei*, the causative organism of African trypanosomiasis or sleeping sickness in humans, is transmitted by the insect vector *Glossina morsitans* (tsetse fly) [30]. The complex life cycle between tsetse flies and their mammalian hosts involves various developmental stages. The blood stream forms known as trypomastigotes in vertebrate hosts are polymorphic, existing as long and slender, short, and stumpy as well as intermediate forms [28]. Metacyclic trypomastigotes injected into human host during a blood meal transform into bloodstream forms. In the vector midgut, bloodstream forms transform into procyclic trypomastigotes, which divide to form epimastigotes, which enter the salivary glands and replicate by binary fission. Previous attempts at retaining infectivity of the long and slender bloodstream forms to mammalian hosts in in vitro cultures were marred by their rapid transformation into noninfective short and stumpy forms resembling those seen in the vector. The long-term maintenance of the bloodstream forms has been a challenge for in vitro culture studies, which was overcome by cultivation of bloodstream trypomastigotes using mammalian feeder layer system in RPMI 1640 medium with 20% heat-inactivated fetal bovine serum and complete life cycle development of *T*. *brucei* has been achieved [49].

Among the first reports of in vitro cultivation of *T*. *brucei* testing various media, the importance of pH toward the multiplication of *T*. *brucei* was stressed. It was concluded that the best results were obtained using MEM 199 buffered with HEPES due to optimal pH conditions (7.4) provided by this medium [57].

Another early publication used bovine fibroblast-like cells in HEPES-buffered Roswell Park Memorial Institute (RPMI)-1640 medium with 20% fetal bovine serum at 37°C where the parasites were able to be maintained for more than 220 days [58]. Moreover, 18 hours after initiation of the primary bovine cell culture, the cell suspension was transferred to a new T-75 culture flask, and fresh medium was added. Sub-culturing was carried out 14 days later when fibroblasts predominated the original culture. The authors recommended to vary the time point of sub-culturing depending on the morphological stages needed as change in medium after 3 days did not lead to the transformation of the slender to stumpy trypanosomes. The parasites obtained in this study were shown to be infective to mammalian hosts.

Other studies also using RPMI-1640 on a bovine embryonic spleen (BESP) feeder layer propagated infective stages of the parasites that were shown to be infective in rats and tsetse flies [59,60]. A novel in vitro system for the cultivation of bloodstream forms of *T*. *brucei* consisted of a feeder layer of fibroblast-like cells derived from embryos of New Zealand white rabbits or the mountain vole *Microtus montanus*, with HEPES-buffered MEM supplemented with Earle’s salts and 15% inactivated rabbit serum [61]. The cultivated bloodstream forms thus obtained were infective to mice and were cyclically transmitted through tsetse flies.

Another approach involved cultivation of *T*. *brucei* in Cunningham’s liquid medium in the presence of head-salivary gland, alimentary tract, and abdominal body wall explants of tsetse flies for up to 66 days. It was shown that after 8 days of cultivation, some of the procyclic forms transformed into metacyclic stages, which were infective to mice [62].

In order to overcome the issue of growth inhibition due to the release of inhibitory metabolites released during culturing of *T*. *brucei* and to avoid constant medium exchanges, Ajoko and Steverding [63] developed a novel method for culturing *T*. *brucei* using transwells placed in standard tissue culture plate wells. Using this approach, a 4-fold increase in the yield of bloodstream forms of the parasites (up to 2 × 10^7^/ml) and an extension of the exponential phase by 1 day compared to previous studies [64] were achieved.

#### *Leishmania* spp.

Leishmaniasis, transmitted by infected female sandflies (*Phlebotomus papatasi*), refers to the disease caused by *Leishmania* spp. According to WHO, leishmaniasis is caused by over 20 species of *Leishmania* and is transmitted by over 90 species of sandflies. It is a severely debilitating neglected tropical disease with outcomes ranging from cutaneous lesions, facial disfigurement to death [30]. The life cycle involves 2 forms: spherical amastigotes found in the mammalian host and motile promastigotes occurring in phlebotomine sandflies, both forms multiply by repeated binary fission [28].

Previous studies reported the use of insect and mammalian cell culture media for large-scale production of promastigotes; however, these techniques were unsuitable for obtaining amastigotes [49]. Several studies starting from the 1980s to the turn of the century cultured *Leishmania* promastigotes in an axenic setting using various media, temperature, and pH conditions [65–68]. Although the promastigotes produced using cell free media were similar to in vivo–derived parasites, there was a shift in research efforts toward using cell lines for culturing *Leishmania* spp. due to loss of virulence of the promastigotes in long-term axenic in vitro cultures.

In spite of the recent advances in cell culture techniques, progress in the field of in vitro culture of *Leishmania* parasites has been slow. Petropolis and colleagues [69] developed a 3D model for studying *Leishmania amazonensis* promastigote migration within a Collagen I (COL I) matrix mimicking the dermal ECM in the host. This study provided valuable insights into the mode of migration within the 3D matrix not feasible in previous experimental cultures. A recent study compared for the first time the 3 most widely used media for cultivation of *Leishmania infantum* promastigotes: Grace’s insect cell culture medium, liver infusion tryptose, and Schneider’s insect medium, without supplementation or supplemented with fetal calf serum and bovine serum albumin [70]. This study concluded that liver infusion tryptose supplemented with fetal calf serum was best suited for growth and maintenance of *L*. *infantum* parasites.

#### Giardia duodenalis

Giardiasis caused by *G*. *duodenalis*, also known as *Giardia lamblia* or *Giardia intestinalis*, is one of the most widespread gastrointestinal ailments worldwide [30]. Despite its global occurrence and severe long-term health consequences especially in children, giardiasis is a neglected tropical disease. The parasitic life cycle includes 2 forms: trophozoites and cysts. After ingestion, cysts hatch into motile trophozoites, which multiply by binary fission and colonize the human intestine [28]. A fundamental challenge for in vitro cultivation of *G*. *duodenalis* used to be the infeasibility of co-culturing parasitic trophozoites and intestinal epithelial cells. Hence, information on host–parasite interaction has been lacking. Most publications have axenically cultured trophozoites either in TYI-S-33 medium [71] or BI-S-33 medium [72] supplemented with bile.

Fisher and colleagues [73] developed a coculture model of trophozoites and Caco-2 cells mimicking the apical–basolateral direction of the small intestinal epithelium. This model using transwell inserts was able to maintain the cocultures for 21 days, thereby providing a viable system for long-term culture of *G*. *duodenalis* in the presence of host cells. This coculture model along with other in vitro cultures of *G*. *duodenalis* published so far would benefit greatly from standardization in order to implement these models across several laboratories [74]. The first report of a 3D coinfection model of *G*. *duodenalis* with *T*. *gondii* in murine organoid–derived monolayers was published recently by Holthaus and colleagues [46]. In order to ensure optimal survival of *G*. *duodenalis* trophozoites while mimicking duodenal conditions, the monolayers were incubated in a modified TYI-S-33 medium rich in cysteine with added bile salts, instead of DMEM-based media. This medium supported the growth of the parasites for over 72 hours in culture.

### Amoebas

#### Entamoeba histolytica

Amebic dysentery, also known as amebiasis, caused by the parasitic amoeba *Entamoeba histolytica* has a worldwide distribution [75]. After malaria, amebiasis is the second leading cause of mortality worldwide attributed to parasitic diseases [76]. The life cycle includes 2 stages: the motile trophozoites and the nonmotile, infective cysts [28]. Transmission occurs alimentarily via consumption of food and water contaminated by fecal material containing mature *E*. *histolytica* cysts.

Optimal growth conditions in vitro for *E*. *histolytica* need to mimic the anaerobic environment of the intestine. Toward this goal, Shah and colleagues [76] compared 2 cultivation methods namely: Anaerocult A (Merck, Darmstadt, Germany) and mineral oil blocking methods of in vitro cultivation of *E*. *histolytica*. The investigators cultivated strains of *E*. *histolytica* under axenic conditions by each of the 2 methods mentioned above. Minimum inhibitory concentration (MIC) of metronidazole activity against the parasites used to measure the efficacy of each culture method showed that both methods were equally reliable for the propagation of *E*. *histolytica*.

**Fig 3:**
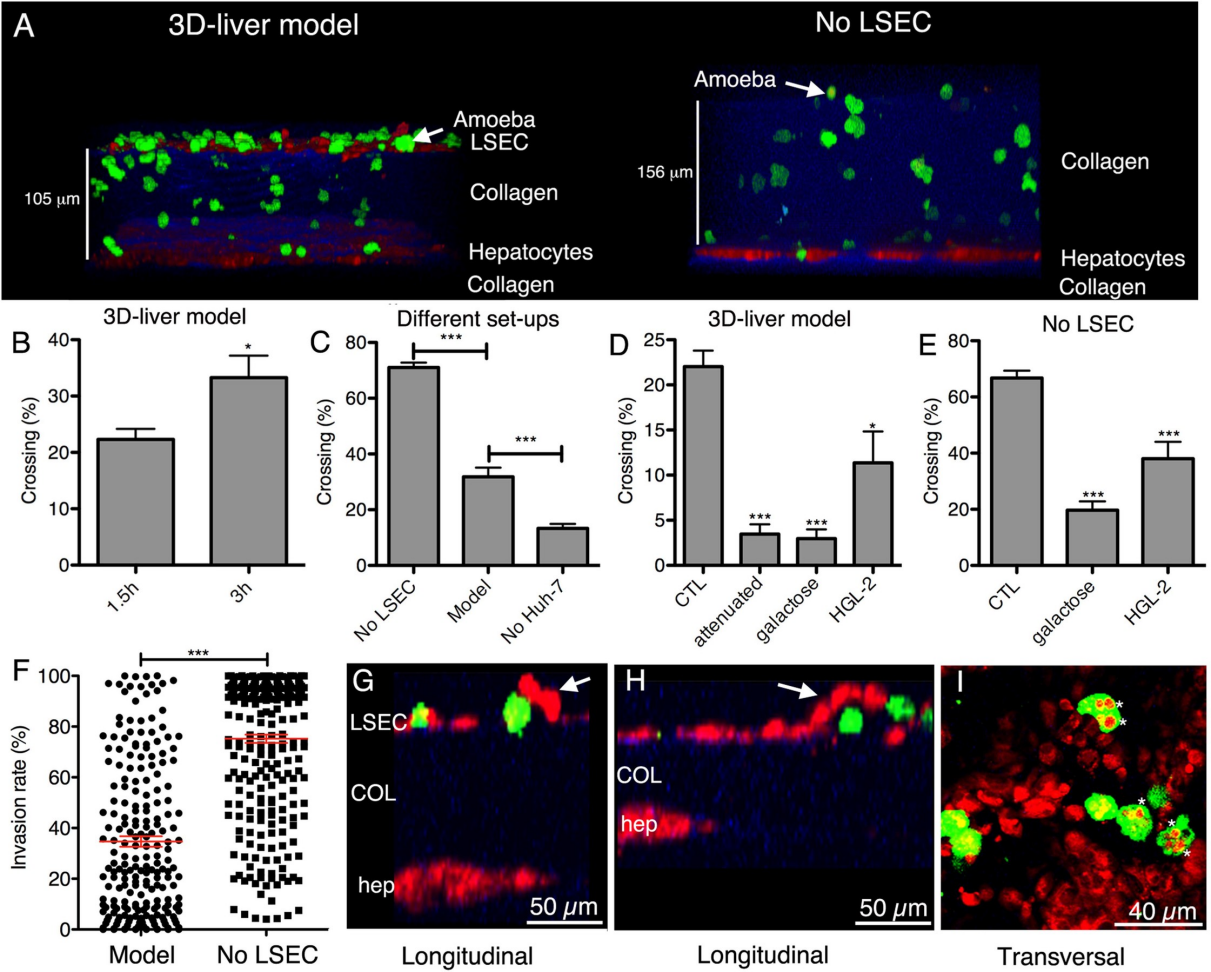
Illustration showing variations of a 3D liver infection model of *E*. *histolytica* infection in vitro. Hepatic cells (red), amoebae (green), and COL-I (collagen-I, blue) are shown. **(A)** Virulent trophozoites added to the medium adhere to the LSEC in the 3D liver model but not in the setup without LSECs (No LSEC) seen in transversal view of reconstructed 3D images. **(B)** Incubation of liver model with trophozoites for 1.5 and 3 hours. **(C)** Incubation of the distinct setups with trophozoites for 3 hours. **(B–E)** Percentage of amoeba crossing the LSEC layer or COL-I matrix. Infection of the liver model **(D)** with various *E*. *histolytica* strains in the presence of glucose/galactose for 1.5 hours and similarly, infection of the setup without LSEC **(F)** for 3 hours. **(F)** Parasite invasion rate at 3 hours for the model and the no LSEC setup showing 0% invasion at the LSEC and 100% at the hepatocyte layer z-position, respectively. Red bars indicate mean values. **(G–I)** Parasite interaction with the LSEC layer of the 3D liver model at 3 hours. In panels (G) and (H), the arrows point to LSEC detaching in the presence of trophozoites. In panel (I), the asterisks point out vesicular structures localized inside the trophozoites labeled with hepatic cell staining. Open-access image reused from [77]. LSEC, liver sinusoidal endothelial cell.

A 3D infection model for amebiasis consisting of cocultures of liver sinusoidal endothelial cells (LSECs) and hepatocytes sandwiched within a collagen-I matrix has been reported (Fig 3) [77]. Although the purpose of this model was to simulate amebic infection of the liver, it could be used as a starting point for further in vitro cultivation studies of this protozoan parasite.

#### Naegleria fowleri

*N*. *fowleri* is the only species of the genus *Naegleria* (free-living amoeboflagellates) known to infect humans. It is a thermophilic amoeboflagellate commonly found in warm fresh water bodies and soils worldwide [28]. The life cycle of *N*. *fowleri* includes trophozoites, motile flagellate forms, and cysts. Infection via trophozoites occurs nasally during recreational activities in infected waters. Within 3 to 7 days after entering through the nose, the parasite crosses the blood–brain barrier leading to primary amebic meningoencephalitis (PAM) affecting the central nervous system [78]. The fatality rate for *N*. *fowleri* infections is over 97%, with only 4 survivors out of 145 known cases in the United States during the period 1962 to 2018 [79].

In one of the earliest publications on in vitro culture of *N*. *fowleri*, Wong and colleagues reported the maintenance of axenic cultures in Nelson’s medium and monkey kidney cell monolayers [80]. However, they observed a loss of virulence of *N*. *fowleri* trophozoites after 50 passages conducted weekly in Nelson’s culture medium (ATCC Medium 710). Gianinazzi and colleagues [81] described an in vitro organotypic slice culture system using rat brain tissues. In comparison to low-dose inoculum, the aforementioned *N*. *fowleri* infection model showed the multiplication of *N*. *fowleri* trophozoites as well as extensive tissue damage in the case of high-dose inoculum (10-fold). However, as pointed out by Coronado-Velazquez and colleagues [82], this model like all other in vitro models has the intrinsic disadvantage when compared to an in vivo model that the physiological conditions in the host cannot be effectively simulated yet. Another disadvantage is the lack of a representative model of a blood–brain barrier.

Burri and colleagues [83] reported in vitro proliferation and cytotoxicity of the trophozoites in various media including Nelson’s medium, PYNFH medium, and PYNFH medium (ATCC Medium 1034) supplemented with liver hydrolysate (LH) in RPMI medium in 24-well plates. To estimate cytotoxicity of *N*. *fowleri*, trophozoites were cocultured directly with the murine fibroblast cell line L929 (ATCC 2148) in a contact-dependent assay, whereas for the contact-independent assay, the trophozoites were added to cell culture inserts (Vitaris, Switzerland). Using the Cytotoxicity Detection kit ^PLUS^ (LDH) (Roche Pharma, Germany) to calculate cytotoxicity, the study concluded that although the addition of LH increased trophozoite proliferation in vitro, there was no correlation of cytotoxicity with the disease potential of the parasites.

Zaongo and colleagues [84] optimized growth conditions for *N*. *fowleri* in vitro by testing several media under varying temperatures and recommended Nelson’s medium supplemented with 1% peptone (N + pep) and modified PYNFH medium without yeast extract to be most suitable. These results were in keeping with previous reports of the affinity of *N*. *fowleri* toward higher temperatures for optimal growth between 30°C and 40°C. With rising global temperatures, the thermophilic nature of this free-living amoeboflagellate is an important consideration when it comes to the effect of climate change in proliferation of this life-threatening organism.

## Discussion

This review highlights current research in the culturing of protozoan parasites associated with high morbidity and mortality. Recent advances in in vitro studies provide new insights into the life cycles of these parasites and contribute to a deeper understanding of pathogenicity potentially leading to more precise and effective treatments and prevention strategies. Current in vitro models provide alternatives to in vivo studies, helping to mimic host–parasite interaction without the need for large-scale animal studies. The protozoan parasites categorized here into apicomplexan, flagellates, and amoebas represent the agents responsible for some of the most debilitating, chronic, and, in some cases, fatal outcomes throughout the world.

In recent years, in vitro models for protozoan candidates such as *Cryptosporidium* spp. and *T*. *gondii* have been well established. In addition to well-recognized agents with a high BoD in humans such as *Plasmodium* spp., this review includes 2 other apicomplexan parasites, *Cryptosporidium* spp. and *T*. *gondii* as well as *G*. *duodenalis* included in the flagellate group. All 3 trypanosomatids included here, *T*. *cruzi*, *T*. *brucei*, *Leishmania* spp., and *Plasmodium* spp. are transmitted by insect vectors and cause debilitating and sometimes lethal diseases especially in the subtropical or tropical regions of the world.

The adverse effects of climate change also have consequences on the emergence of pathogens in regions far beyond endemic areas. *N*. *fowleri*, for example, thrives in warm waters, and, as global temperatures rise, it is predicted that the incidence of PAM would increase considerably [85]. According to WHO, *Leishmania* is another climate-sensitive parasite with changes in the environment directly affecting its epidemiology. Climate change has a direct effect on the increased incidence of insect vector-borne parasitic diseases such as those caused by *Plasmodium* spp. and *Trypanosoma* spp. [53] that are more prevalent in the developing world but are now being reported in industrialized nations likely due to migration and reemergence of these diseases. For instance, adverse weather events such as prolonged periods of heavy rainfall and milder winters have been known to aid the spread of malarial mosquitos [86]. Robust in vitro models would aid in the application of modern research tools such as transcriptomics to study protozoan pathogens.

Due to the qualitative appraisal of the current literature in this review, particular care was taken in the selection of papers to provide a representative picture of the research published on in vitro models. Publications highlighting significant advancements and turning points in the study of each of the parasites were included. Even within the in vitro models available, there exists a broad variation in terms of complexity of the model system; resources needed; cell lines used as well as reproducibility. Advanced 3D in vitro models already exist for certain well-known parasites. In the case of *Cryptosporidium* spp., for instance, while some publications have reported using stem-cell derived air–liquid interface monolayers [36], others have demonstrated more complex models employing a 2D cell culture platform using COLO-680N cells [40] and a 3D organoid culture using hollow fiber technology [41] for the cultivation of parasites in the laboratory. On the other hand, there is a lack of funding and interest in the scientific community for other lesser known yet severe parasitic diseases to humans mainly affecting the developing regions of the world.

The in vitro models summarized in this review are at various stages of development, with some being highly sophisticated 3D cultures, while others are simpler models with less progress made in the past decades. In summary, in vitro models offer the following advantages in the fight against complex parasitic infections: ready availability of various parasitic life cycle stages for further research; platforms for testing antiparasitic drugs and potential vaccines; provision of material for the exploration of pathophysiology and genetics; and alternatives to the ethical implications of using laboratory animals for long-term in vivo studies. Furthermore, organoid-based 3D models are amenable to high-throughput use, live cell imaging, and transcriptome analyses [46].

Global climate change and other anthropogenic stressors such as migration and wildlife trade contribute to the spread of zoonoses and disease vectors, thereby placing an urgency on the need for effective intervention strategies for the control of zoonotic parasitic diseases [53,87,88]. In vitro models are valuable tools toward in-depth studies of parasite biology simulating in vivo conditions in patients. The model systems detailed here may serve to provide ideas for in vitro culturing of other protozoan parasites not included in this review. For instance, due to their common ancestry, apicomplexan parasites show many similarities in genetics, molecular biology and pathogen-specific cellular processes [26]. Advanced 3D culture models elucidating the processes involved in parasite replication leading to host cell and tissue damage are essential for effective disease prevention strategies.

## Future perspectives

The long-standing use of cell monolayers for growth and maintenance of protozoan parasites in vitro is gradually being advanced by novel 3D technologies. Three-dimensional models such as organoids and spheroids serve as bridges between animal models and the microenvironment within the human host. Stem cell–derived intestinal organoids simulate natural host conditions and aid in the study of host–pathogen interaction. Continued advances in 3D cultures open up new avenues for investigating the immunological basis of chronicity, dormancy and asymptomatic disease progression. Standardization and harmonization of culture conditions for protozoa in in vitro settings would aid in drug development and vaccine trials for debilitating diseases affecting communities worldwide.

**Key learning points**

- Protozoan parasites cause severe, debilitating, and fatal diseases worldwide.
- Culturing protozoan parasites in vitro has been challenging due to their complex life cycles involving various stages. In vivo studies pose ethical considerations and are not always representative systems for human hosts.
- In recent years, novel studies including 3D models have been published for culturing protozoa in vitro.
- Climate change and socioeconomic factors such as migration emphasize the need to study the disease cycles in order to develop effective prevention and treatment strategies.
- This review aims to give a comprehensive overview of current in vitro models for culturing protozoan parasites.

**Top five papers**

1. Wilke G, Funkhouser-Jones LJ, Wang Y, Ravindran S, Wang Q, Beatty WL, et al. A Stem-Cell-Derived Platform Enables Complete Cryptosporidium Development In Vitro and Genetic Tractability. Cell Host Microbe. 2019;26(1):123–34.e8.
2. Derricott H, Luu L, Fong WY, Hartley CS, Johnston LJ, Armstrong SD, et al. Developing a 3D intestinal epithelium model for livestock species. Cell Tissue Res. 2019;375(2):409–24.
3. Chua ACY, Ananthanarayanan A, Ong JJY, Wong JY, Yip A, Singh NH, et al. Hepatic spheroids used as an *in vitro* model to study malaria relapse. Biomaterials. 2019;216:119221.
4. Rodriguez ME, Rizzi M, Caeiro L, Masip Y, Sanchez DO, Tekiel V. Transmigration of *Trypanosoma cruzi* Trypomastigotes through 3D Spheroids Mimicking Host Tissues. Methods Mol Biol. 2019;1955:165–77.
5. Zaongo SD, Shaio MF, Ji DD. Effects of Culture Media On *Naegleria fowleri* Growth At Different Temperatures. J Parasitol. 2018;104(5):451–6.
